# Increased SLC7A3 Expression Inhibits Tumor Cell Proliferation and Predicts a Favorable Prognosis in Breast Cancer

**DOI:** 10.2174/0115748928279007231130070056

**Published:** 2024-01-08

**Authors:** Lifang He, Yue Xu, Jiediao Lin, Stanley Li Lin, Yukun Cui

**Affiliations:** 1 Guangdong Provincial Key Laboratory for Breast Cancer Diagnosis and Treatment, Cancer Hospital of Shantou University Medical College, Shantou, Guangdong Province, 515000, China;; 2 Breast Center, Cancer Hospital of Shantou University Medical College, Shantou, Guangdong Province, 515000, China;; 3 Department of Cell Biology and Genetics, Shantou University Medical College, Shantou, Guangdong Province, 515000, China

**Keywords:** SLC7A3, arginine, tumor suppressor, breast cancer, cell proliferation, prognosis

## Abstract

**Background:**

Arginine plays significant and contrasting roles in breast cancer growth and survival. However, the factors governing arginine balance remain poorly characterized.

**Objective:**

We aimed to identify the molecule that governs arginine metabolism in breast cancer and to elucidate its significance.

**Methods:**

We analyzed the correlation between the expression of solute carrier family 7 member 3 (SLC7A3), the major arginine transporter, and breast cancer survival in various databases, including GEPIA, UALCAN, Metascape, String, Oncomine, KM-plotter, CBioPortal and PrognoScan databases. Additionally, we validated our findings through bioinformatic analyses and experimental investigations, including colony formation, wound healing, transwell, and mammosphere formation assays.

**Results:**

Our analysis revealed a significant reduction in SLC7A3 expression in all breast cancer subtypes compared to adjacent breast tissues. Kaplan-Meier survival analyses demonstrated that high SLC7A3 expression was positively associated with decreased nodal metastasis (HR=0.70, 95% CI [0.55, 0.89]), ER positivity (HR=0.79, 95% CI [0.65, 0.95]), and HER2 negativity (HR=0.69, 95% CI [0.58, 0.82]), and increased recurrence-free survival. Moreover, low SLC7A3 expression predicted poor prognosis in breast cancer patients for overall survival. Additionally, the knockdown of SLC7A3 in MCF-7 and MDA-MB-231 cells resulted in increased cell proliferation and invasion *in vitro*.

**Conclusion:**

Our findings indicate a downregulation of SLC7A3 expression in breast cancer tissues compared to adjacent breast tissues. High SLC7A3 expression could serve as a prognostic indicator for favorable outcomes in breast cancer patients due to its inhibitory effects on breast cancer cell proliferation and invasion.

## 1 INTRODUCTION

Breast cancer represents the most common malignant tumor among women and exhibits significant heterogeneity, posing a major challenge for accurate diagnosis and effective therapy [1, 2]. While conventional histopathological classification provides prognostic insights, molecular-level biomarkers can enhance prognostic accuracy and inform targeted therapeutic strategies [2-4]. The widely used breast cancer tumor marker CA15-3 holds clinical significance in monitoring breast cancer recurrence and metastasis. Nonetheless, its limited sensitivity and inadequate detection rate hinder its utility for early breast cancer detection. Thus, the pursuit of highly sensitive biomarkers for early breast cancer diagnosis through research and development is constantly required for timely diagnosis and treatment of the disease and to decrease its recurrence and metastasis [5, 6].

Arginine is a vital component of the arginine-glutamate cycle, which is essential for the synthesis of downstream metabolites, such as nitric oxide (NO), polyamines, and creatine, that are crucial for cell proliferation and survival [7-9]. While arginine is considered nonessential, it can become essential during periods of metabolic or traumatic stress when the endogenous arginine supply is insufficient to meet physiological demands [10]. Intriguingly, arginine exhibits diverse roles in cancers. It inhibits tumor growth by itself, yet its role in NO synthesis can both enhance or inhibit tumor development [10]. Additionally, arginine serves as an effector for mTOR1 activation in response to glutamine starvation, promoting cell growth [10], but arginine deprivation can lead to cancer cell death [11].

The solute carrier (SLC) proteins constitute the second-largest membrane protein family, encompassing 458 transporter proteins distributed across 65 families [12]. They facilitate the transport of diverse small molecules through cell membranes, regulating fundamental physiological processes from nutrient uptake to drug disposition [13]. Increasing evidence underscores the pivotal role of SLC upregulation and/or downregulation in the onset and progression of human malignancies. Notably, endogenous SLC substrates encompass estrogen and its conjugates, playing significant roles in hormone-dependent cancers [14]. SLC7A3 is an arginine transporter, a recent study revealed that breast cancer patients with high SLC7A4 expression had better prognoses, while the role of SLC7A3 in breast cancer progression remains unclear [15-17]. Lanzavecchia *et al*. highlighted the critical role of intracellular arginine in regulating cell survival and anti-tumor activity, influencing T cell metabolism and improving survival and anti-tumor responses [18]. Host autophagy gene deletion has been associated with reduced arginine cycling, offering novel therapeutic avenues in cancer [19]. Considering SLC7A3's role as an arginine transporter and its connection to tumorigenesis [20-22], The substantial functional overlap among SLC transporters and the complexity of their interplay in response to environmental stresses remain poorly elucidated, which poses challenges in understanding the specific roles and therapeutic potential of SLC7A3 in breast cancer [23]. The precise mechanisms underlying the association between SLC7A3 expression and survival outcomes in breast cancer patients are yet to be thoroughly explored. There's a call for further studies to verify the diagnostic and prognostic value of SLC7A3 in breast cancer, indicating a need for more comprehensive research to substantiate initial findings [17]. In this study, we explored the diagnostic and prognostic value of SLC7A3 in breast cancer.

## 2 MATERIALS AND METHODS

Fig. (**1**) illustrates the research methodology for the present investigation.

### 2.1 UALCAN

UALCAN (http://ualcan.path.uab.edu/) represents a web-centric utility offering a comprehensive examination of data sourced from The Cancer Genome Atlas (TCGA) and MET500 transcriptome databases [24]. By utilizing UALCAN, an exploration into the expression of SLC7A3 and its correlation with diverse clinicopathological factors of breast cancer, including gender, cancer stage, nodal metastasis status, age, race, and TP53 mutation status, were conducted.

### 2.2 Gene Expression in Profiling Interactive Analysis (GEPIA)

GEPIA 2.0 (http://gepia.cancer-pku.cn/index.html) is a user-friendly web platform designed for gene expression analysis, leveraging data from both TCGA and the GTEx (Genotype-Tissue Expression) database [25]. The GTEx database specifically focuses on collecting and sequencing tissue samples exclusively from healthy individuals. For this particular investigation, TCGA-breast cancer data was utilized to examine the expression pattern of SLC7A3. To facilitate the analysis, the “Expression DIY” module of GEPIA integrated TCGA data from normal tissue with corresponding data from GTEx. A log2 transformation of the expression values (TPM - Transcripts per million) plus one was applied to establish a log scale for comparing the expression of SLC7A3 between breast cancer samples and adjacent normal breast tissue samples.

### 2.3 Oncomine

Oncomine (www.oncomine.org) is a gene chip-oriented database which can be used for the exploration of gene transcription expression in diverse cancer types through data mining [26]. In this study, Oncomine was employed to investigate the mRNA level of SLC7A3 in breast cancer. The threshold parameters utilized were a p-value of 0.05, a fold-change of 1.5, and an inclusion of all gene ranks.

### 2.4 CBioPortal

The cBioPortal for Cancer Genomics houses extensive datasets of cancer genomes, offering functionalities for visualization, download, and analysis [27]. In this study, we opted for a dataset comprising 1084 cases of breast cancer, which was subjected to in-depth analysis using cBioPortal. The “OncoPrint” and “Cancer Types Summary” modules were employed to scrutinize the types and frequencies of genomic alterations concerning SLC7A3 in breast cancer. Additionally, the “Comparison/Survival” module of cBioPortal was utilized to examine the overall survival (OS) and disease-free survival (DFS) associated with SLC7A3.

### 2.5 Metascape Analysis

Metascape is an intuitive tool designed for gene annotation and gene set enrichment analysis [28]. In this study, we evaluated the functionalities of genes associated with SLC7A3. The enrichment analysis criteria were set with a p-value threshold of 0.01, an enrichment coefficient greater than 1.5, and a minimum value of 3. By utilizing Metascape's GO and KEGG analysis modules, we delved into the biological processes (BPs), cellular components (CCs), and molecular functions (MFs) of SLC7A3-associated genes in breast cancer.

### 2.6 TIMER Database Analysis

TIMER 2.0 (https://cistrome.shinyapps.io/timer/) is an interactive online platform that facilitates extensive examination of immune cell infiltration levels. The TIMER database encompasses a vast collection of 10987 samples spanning 32 cancer types obtained from TCGA. In this research, we utilized the “Diff Exp” module within TIMER to investigate the expression of SLC7A3 across various types of cancers [29].

### 2.7 Kaplan-Meier (KM) Plotter Database Analysis

We assessed the prognostic significance of SLC7A3 in breast cancer using the online KM Plotter database (http://kmplot.com), which provided gene expression data and survival information of 4934 clinical breast cancer patients. The patients were categorized into two groups based on the median expression level (high expression and low expression). Subsequently, we determined the overall survival (OS), progression-free survival (PFS), and post-progression survival (PPS) by calculating hazard ratios (HRs) along with their corresponding 95% confidence intervals (95% CIs) and log-rank p-values. To identify the clinicopathological parameters of SLC7A3 that showed differential expression in breast cancer, we utilized the “forest plot” package in R software.

### 2.8 Analysis of SLC7A3-Interacting Genes and Proteins

We utilized the GeneMANIA database (http://www.genemania.org) to construct an interactive network for SLC7A3. Additionally, a protein-protein interaction (PPI) network was established using the STRING online database (https://string-db.org/). The STRING database incorporates all known and predicted associations between proteins, encompassing both physical interactions and functional associations.

### 2.9 Immunohistochemistry (IHC) Staining

This research received approval from the Research Ethics Committee of the Cancer Hospital affiliated with Shantou University Medical College. Prior to the study, written informed consent was obtained from all participants. For immunohistochemical staining, a total of thirty-one cases of formalin-fixed, paraffin-embedded breast cancer tissues and adjacent breast tissues were used. Briefly, 4-μm tissue slices were placed on glass microscope slides and subjected to deparaffinization in xylene, followed by rehydration in increasing dilutions of alcohol. Citrate antigen unmasking was carried out in a high-temperature water bath, and the slices were then cooled and rinsed. 3% hydrogen peroxide was used to quench endogenous peroxidases. After three washes with PBS, the sections were incubated with goat serum (Boster Biological Technology, USA, California Tech Culture Center, California, cat#AR0009) for ten minutes to block nonspecific binding. Next, the sections were incubated overnight at 4°C with the primary rabbit SLC7A3 polyclonal antibody (1:200, Invitrogen, USA, Waltham, Massachusetts, cat#PA5-51978). On the following day, the sections were washed three times with PBS and then incubated with the secondary antibody at room temperature for 30 minutes (Gene Tech Shanghai Company Limited, China, Shanghai, cat#GK500510A). Immunoperoxidase staining was performed using a DAB color kit (Maixin Biotechnology, China, Guangzhou, cat#DAB-0031). Haematoxylin was used for counterstaining before the coverslip was applied, and the samples were examined by light microscopy. The immunohistochemical staining results were analyzed and scored by two pathologists who were unaware of the sources of the clinical samples. Staining intensity was evaluated using a semi-quantitative integration method, with intensity scores assigned as follows: 0 for negative, 1 for weak, 2 for moderate, and 3 for strong. The frequency of positive cells was categorized as follows: 0 for less than 5%, 1 for 5% to 25%, 2 for 26% to 50%, 3 for 51% to 75%, and 4 for greater than 75%. A combined “histoscore” was calculated as the result of multiplying the average staining intensity (0-3) by the average percentage of positive cells (0-4), with a maximum possible score of 12. The degree of SLC7A3 staining was determined by summing the staining intensity and the positive cell percentage scores, establishing levels of low expression (0-6) and high expression [30].

### 2.10 Cell Culture

Human breast cancer cell lines MDA-MB-231 and MCF-7 were purchased from the American Type Culture Collection. All cell lines were cultured in Dulbecco's modified Eagle's medium (DMEM, Gibco, USA, New York) supplemented with 10% fetal bovine serum (FBS). Incubation of all cells was carried out in a CO_2_ incubator at 37°C with 5% CO_2_.

### 2.11 Transient Transfection

GenePharma (Shanghai, China) provided small interfering RNA (siRNA) targeting SLC7A3 and a control siRNA (Table **S1**). Transfection of MCF-7 and MDA-MB-231 cells was performed using 80nM of SLC7A3 siRNA and control siRNA, respectively, for 36 hours. Lipofectamine 3000 reagent (Invitrogen, USA, Waltham, Massachusetts, cat#L3000015) was used for transfection following the manufacturer's instructions. Subsequently, SLC7A3 gene expression analysis and relevant functional assays were performed.

### 2.12 Western Blot Analysis

Proteins were extracted using RIPA lysis buffer (Solarbio, China, Beijing, cat#0020), and the protein concentration was determined using a BCA Protein Assay Kit (Solarbio, China, Beijing, cat#PC0020). Subsequently, 50μg of proteins were separated on 12% SDS-PAGE gels (Solarbio, China, Beijing, cat#P1200) and transferred onto PVDF membranes (Rainbio, China, Shanghai) using a transfer system (Bio-Rad). To block non-specific binding, the membranes were incubated with 5% bovine serum albumin (Solarbio, China, Beijing, cat#A8020) for 1 hour, followed by overnight incubation with primary antibodies at 4°C. The primary antibodies used were anti-SLC7A3 (1:500, Thermo Fisher, USA, Waltham, Massachusetts, cat#PA5-51978), and anti-GAPDH (1:20000, Abcam, UK, Cambridge, cat#ab128915). After washing the membranes with TBST, they were further incubated with goat anti-rabbit HRP-conjugated secondary antibody (1:20000, Abcam, UK, Cambridge, cat#ab97051). Signal detection was accomplished using an ECL Western blotting Kit (Thermo Fisher, USA, Waltham, Massachusetts, cat#34580) and Bio-Rad XR Image Analysis System (Bio-Rad, USA, California).

### 2.13 Real-time Polymerase Chain Reaction (RT-PCR)

Total RNA was extracted from the samples using TRIzol (Invitrogen, USA, Waltham, Massachusetts, cat#343911) following the manufacturer's protocol. The RNA was then reverse transcribed into cDNA using an RT-PCR kit (Takara, Japan, Otsu, cat#RR047A and RR820A). β-Actin was used as a loading control. The real-time PCR reactions were carried out using the 7300 Real-Time PCR System (Applied Biosystems, USA, Waltham), and the resulting data were presented as 2^-△△Ct^ values. Each experiment was performed three times to ensure reproducibility. The sequence of primers used was provided in Table **S2**.

### 2.14 Cell Counting Kit-8 (CCK-8)

To assess the viability of MCF-7 and MDA-MB-231 cells, both cell lines were seeded into 96-well plates at a density of 1000 cells per well. Subsequently, CCK-8 solution (Beyotime, China, Zhengzhou, Henan, cat C0039) was added to each well at the specified time points. Following a 2.5-hour incubation at 37°C with 5% CO_2_, the absorbance (OD value) was measured at a wavelength of 450nm using a Thermo K3 plate reader (Thermo Fisher, USA, Waltham, Massachusetts). All OD values were then subjected to analysis using Image J software.

### 2.15 Wound Healing

MCF-7 and MDA-MB-231 cells were seeded in 6-well plates at a density of 4×10^5^ cells per well. A uniform wound was created by scratching the cell monolayer using a 200μL plastic pipette tip. After 24 hours of incubation, the wound width was observed under a microscope, and photographs of at least 3 random fields were captured. The cell migration capability was quantified by measuring the closure of the gap distance, which was accomplished using Image J software.

### 2.16 Colony Formation

For assessing the viability of MCF-7 and MDA-MB-231 cells following SLC7A3 silencing, 800 cells were plated into individual wells of 6-well plates. The cells were then cultured for 15 days until visible colony formation occurred. Subsequently, the colonies were fixed with methanol for 20 minutes and stained with 0.4% crystal violet(Solarbio, China, Beijing, cat#C8470) before being counted.

### 2.17 Transwell Assay

To investigate cell migration and invasion post SLC7A3 silencing, 5×10^4^ MCF-7 and MDA-MB-231 cells were employed. The invasion assay was carried out using a 6.5-mm diameter transwell chamber equipped with an 8-μm pore size polycarbonate membrane insert (Corning, USA, New York, cat#353097). Matrigel (Corning, USA, New York, cat#356234) was diluted 1:7 with serum-free medium and used to coat the upper chamber side of the transwell membrane. After allowing migration for 24 hours, cells within the chambers were fixed with 4% paraformaldehyde, followed by staining with 0.1% crystal violet (Solarbio, China, Beijing, cat#C8470). Cell counts were performed in quadruplicate fields of view under 100×magnification.

### 2.18 Mammosphere Formation

A total of one thousand MCF-7 or MDA-MB-231 cells were seeded into 6-well ultra-low attachment plates (Corning, USA, New York, cat#3471) with 5ml mammary epithelial growth medium, supplemented with 5% FBS, 20ng/ml EGF (Thermo Fisher, USA, Waltham, Massachusetts, cat#PHG0314), and 10ng/ml bFGF (Thermo Fisher, USA, Waltham, Massachusetts, cat#PHG0367). Two weeks later, images of mammospheres were taken using a microscope.

### 2.19 Statistical Analysis

The Kaplan-Meier plots and GEPIA results were presented with hazard ratios (HR) and p-values or Cox p-values calculated using the log-rank test. To assess the correlation of gene expression, Spearman's correlation coefficient and its statistical significance were employed. A correlation heat map depicting the relationship between SLC7A3 and its associated genes was generated using data from the UALCAN database with Spearman correlation analysis. A p-value of less than 0.05 was considered statistically significant. All experiments were conducted three times, with samples measured in duplicate or triplicate. Statistical differences were evaluated using a two-tailed t-test. Results are expressed as mean±standard deviation (SD), unless specified otherwise. For statistical analysis, GraphPad Prism 8.0 software (GraphPad Software, San Diego, CA) was used.

## 3 RESULTS AND DISCUSSION

### 3.1 SLC7A3 Expression is Decreased in Breast Cancer Patients

**Fig. (2):**
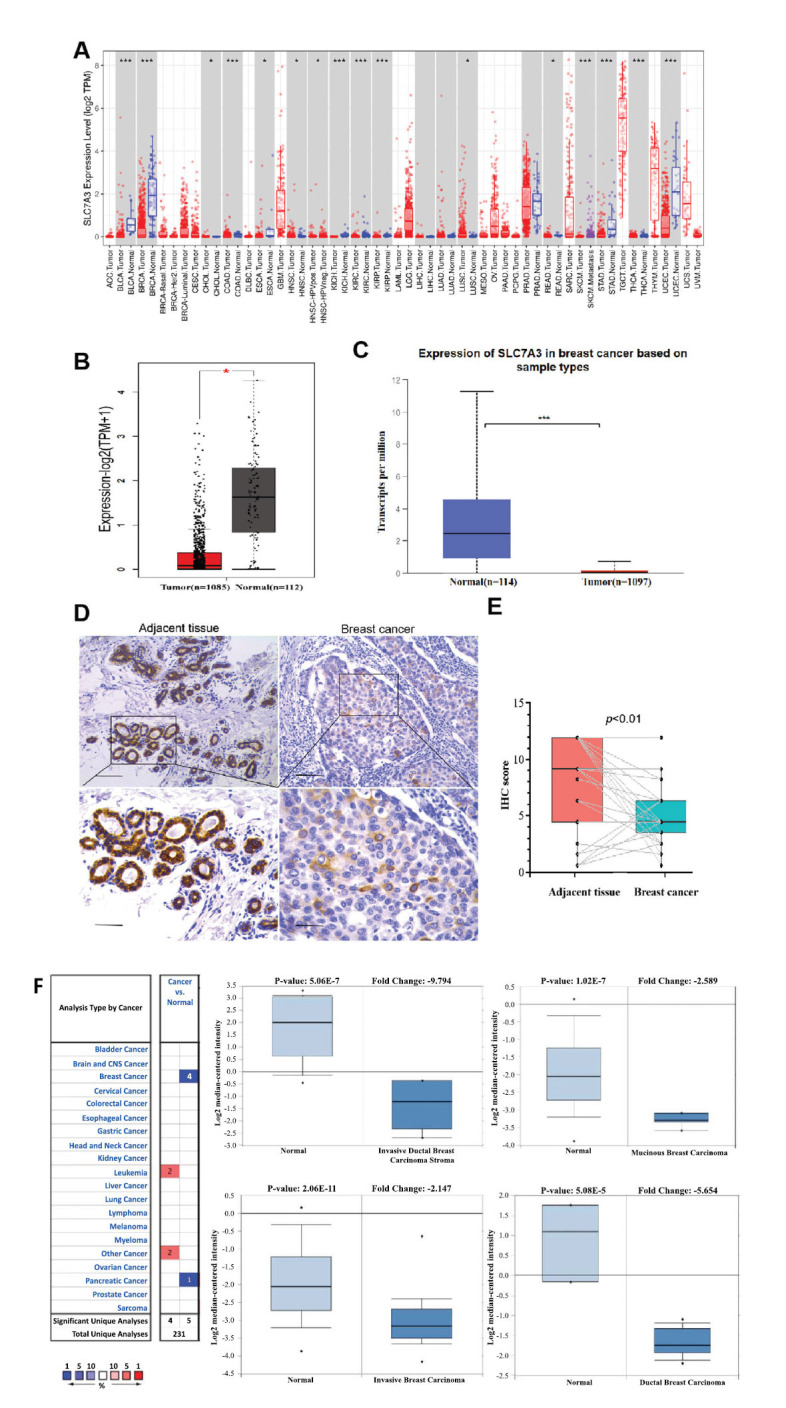
Expression of SLC7A3 in cancer. **(A)** SLC7A3 expression in different types of cancer was investigated using the TIMER database. **(B)** Decreased expression of SLC7A3 in breast cancer compared to normal tissues in the GEPIA database. **(C)** SLC7A3 expression in breast cancer was examined by using the UALCAN database. **(D)** Immunohistochemical staining of SLC7A3 was performed in ER-positive breast cancer and normal breast tissues. Representative images are shown. Scare bars, 50μm. **(E)** Analysis of SLC7A3 expression in ER-positive breast cancer and adjacent normal tissues based on IHC score. **(F)** SLC7A3 expression in breast cancer and normal tissues. *p<0.05, **p<0.01, ***p<0.0001.

First, we compared SLC7A3 mRNA expression in various human cancer types using the online Tumor Immune Estimation Resource database (TIMER). In general, tumor tissues displayed lower SLC7A3 expression than corresponding normal tissues, including bladder urothelial carcinoma, breast invasive carcinoma, stomach adenocarcinoma, and uterine corpus endometrial carcinoma. Among these cancer types, breast tumors exhibited the most significant differences, indicating a potentially prominent role of SLC7A3 in breast cancer (Fig. **2A**). Using the GEPIA database, we further observed substantially lower SLC7A3 mRNA expression in breast cancer tissues compared to normal breast tissues (Fig. **2B**). Additionally, direct analysis of SLC7A3 expression in breast cancer and adjacent tissues using data from the UALCAN database, based on The Cancer Genome Atlas (TCGA), confirmed a significant decrease in SLC7A3 expression in breast cancer tissues (Fig. **2C**). These findings collectively indicate a reduction in SLC7A3 expression in breast cancer, suggesting its potential important regulatory role in the progression of breast cancer.

Immunohistochemical staining for SLC7A3 was conducted to confirm its expression in breast cancer tissues further. The level of SLC7A3 was visibly lower in breast invasive ductal carcinoma tissues compared to normal breast tissues (Fig. **2D**) and showed a significant reduction in 31 pairs of tumor samples compared with adjacent normal samples (Fig. **2E**). We also utilized the Oncomine database to analyze the expression of SLC7A3 mRNA in cancer and normal clinical samples, as shown in Table **1**. A total of 317 datasets, including 49,244 samples, were selected based on the criteria of a p-value less than 1X10^-4^ and fold change greater than 2. The results revealed that SLC7A3 was expressed at low level in both breast and pancreatic cancers compared to normal tissues (Fig. **2F**).

### 3.2 SLC7A3 Expression and Clinicopathological Characteristics of Breast Cancer Patients

**Fig. (3):**
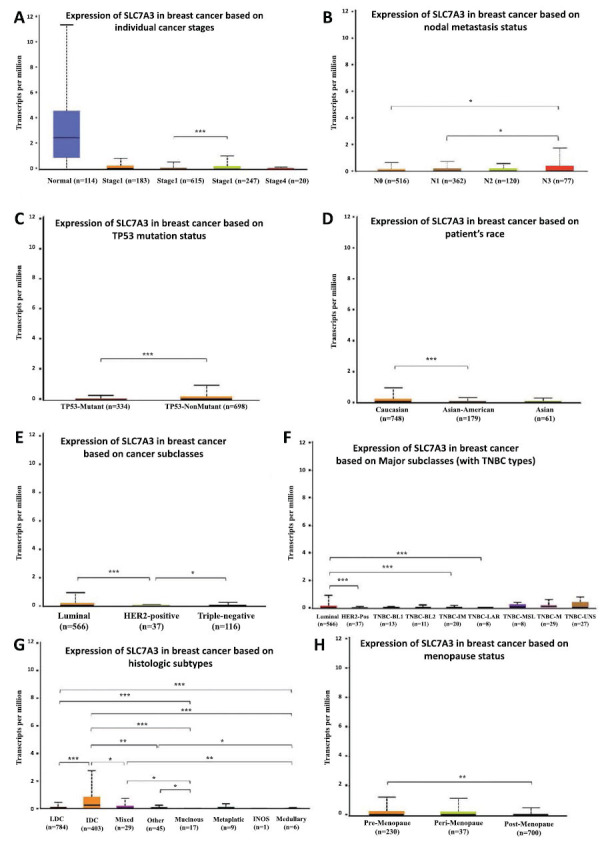
Box plots evaluating SLC7A3 expression among different groups of patients based on clinical parameters using the UALCAN database. Analysis is shown for cancer stages **(A)**, nodal metastasis status **(B)**, TP53 mutation status **(C)**, patient race **(D)**, breast cancer subclasses **(E)**, TNBC type **(F)**, pathological subtypes **(G)**, and menopause status **(H)**. **Abbreviations:** BRCA: breast cancer; N0: no regional lymph node metastasis; N1: metastases in 1 to 3 axillary lymph nodes; N2: metastases in 4 to 9 axillary lymph nodes; N3: metastases in 10 or more axillary lymph nodes. TNBC-BL1: TNBC basal-like 1; TNBC-BL2: TNBC basal-like 2; TNBC-IM: TNBC immunomodulatory; TNBC-M: TNBC mesenchymal; TNBC-MSL: TNBC mesenchymal stem-like; TNBC-LAR: TNBC luminal androgen receptor; TNBC-UNS: TNBC unspecified. IDC: infiltrating ductal carcinoma; ILC: infiltrating lobular carcinoma; Mixed: mixed histology; Mucinous: mucinous carcinoma; Medullary: medullary carcinoma; Metaplastic: metaplastic carcinoma; INOS: infiltrating carcinoma not otherwise specified. *p<0.05, **p<0.01, ***p<0.001.

Using the UALCAN online tool, we investigated the expression of SLC7A3 in different patient groups based on various clinicopathological parameters. Based on breast sample types, SLC7A3 expression was lower in primary breast cancer samples compared to normal tissues (Fig. **2C**). A decrease in the expression of SLC7A3 was observed in breast cancer patients across stages 1, 2, 3, and 4, compared to normal controls (Fig. **3A**). For breast cancer with nodal metastasis, where SLC7A3 expression was lower in patients with breast cancer classified as N0, N1, or N2, compared to N3 (Fig. **3B**).

Given that SLC7A3 is a p53-inducible gene involved in the metabolic stress response [11], we examined its correlation with the expression of wild-type or mutant TP53. Both TP53 wild-type and TP53-mutant breast cancer patients showed a reduction in SLC7A3 expression compared with normal controls, with significantly lower SLC7A3 expression in TP53-mutant breast cancer patients (Fig. **3C**). However, SLC7A3 levels were also lower in TP53-wild-type tumors, suggesting TP53 status is not the primary determinant of SLC7A3 levels in breast cancer. Furthermore, SLC7A3 expression was decreased in breast cancer patients of Caucasian, Asian, and African-American ethnicity, with higher SLC7A3 levels observed in Caucasian patient tumors than in their African-American and Asian counterparts (Fig. **3D**). Regarding breast cancer subtypes, HER2-positive tumors displayed a generally greater reduction in SLC7A3 expression compared to TNBC tumors and luminal breast cancer tumors (Fig. **3E**). Among the major subclasses, luminal tumors exhibited higher SLC7A3 expression than TNBC-luminal androgen receptor, HER2-positive, and TNBC tumors (Fig. **3F**). SLC7A3 expression in infiltrating lobular carcinoma was higher than in most other pathological types of breast cancer (Fig. **3G**), and SLC7A3 expression was higher in pre-menopausal tumors than in post-menopausal tumors (Fig. **3H**). These results indicate significant variations in SLC7A3 expression among different subtypes of breast cancers.

### 3.3 Reduced SLC7A3 Expression is Associated with Poor Prognosis in Early-stage Breast Cancer Patients

**Fig. (4):**
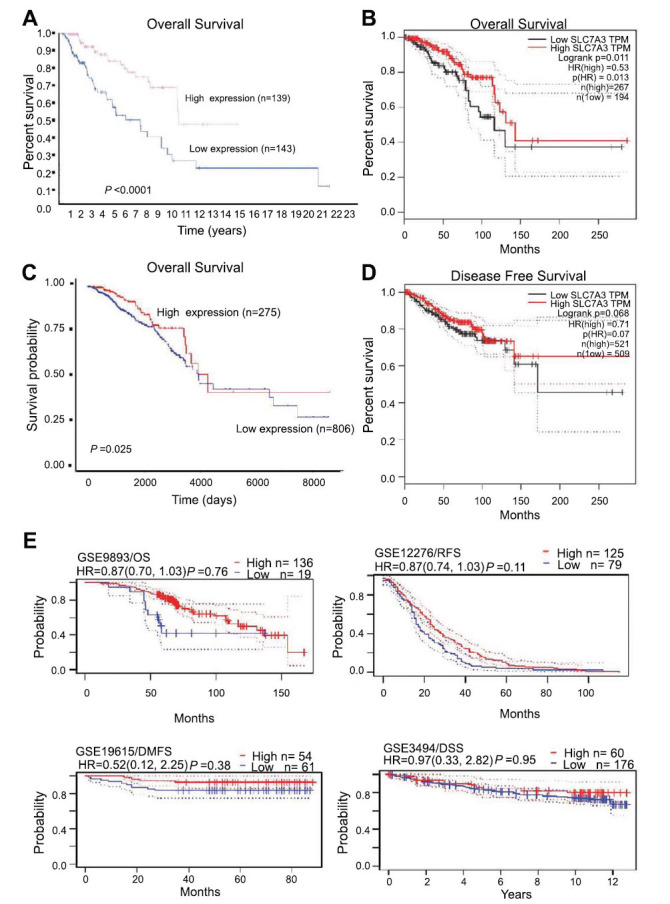
Survival curves evaluating the prognostic value of SLC7A3. (**A**) OS curves using the Human Protein Atlas. (**B**) OS curves using the GEPIA database. (**C**) OS curves using the UALCAN database. (**D**) DFS curves using the GEPIA database. (**E**) OS, RFS, DSS and DMFS curves using the PrognoScan database. **Abbreviation:** BRCA: breast cancer. All dotted lines represent confidence bands.

Based on the above results, we investigated whether SLC7A3 functions as a tumor suppressor during breast cancer progression. The close association between low SLC7A3 expression and breast cancer progression and metastasis prompted us to examine the prognostic value of SLC7A3 gene expression. Breast cancer patients with lower SLC7A3 expression, as determined by the median FPKM (fragments per kilobase million) value of TCGA data, exhibited a worse overall survival (OS) according to the Human Protein Atlas Kaplan-Meier analysis module (Fig. **4A**). In the GEPIA database, which utilizes both GTEx and TCGA data, the prognosis based on SLC7A3 expression was found to depend on the time after diagnosis. Patients with higher expression of SLC7A3 had a better OS, according to the results of follow-up analysis (Fig. **4B** and **C**). Additionally, Fig. (**3D**) showed that patients with high SLC7A3 expression displayed a tendency toward longer disease-free survival probability. These survival curves indicate that SLC7A3 expression level was associated with the prognosis of breast cancer patients and warrants further analysis. To evaluate potential tumor markers and therapeutic targets, we used the PrognoScan database to study the relationship between gene expression and patient outcomes. The results revealed that breast cancer patients with high SLC7A3 expression had greater overall survival (OS), relapse-free survival (RFS), disease-specific survival (DSS), and distant metastasis-free survival (DMFS) compared to patients with low SLC7A3 expression, suggesting that SLC7A3 could act as a protective factor in breast cancer (Fig. **4E**).

### 3.4 Validation of the Prognostic Value of SLC7A3 based on Multiple Clinicopathological Features

**Fig. (5):**
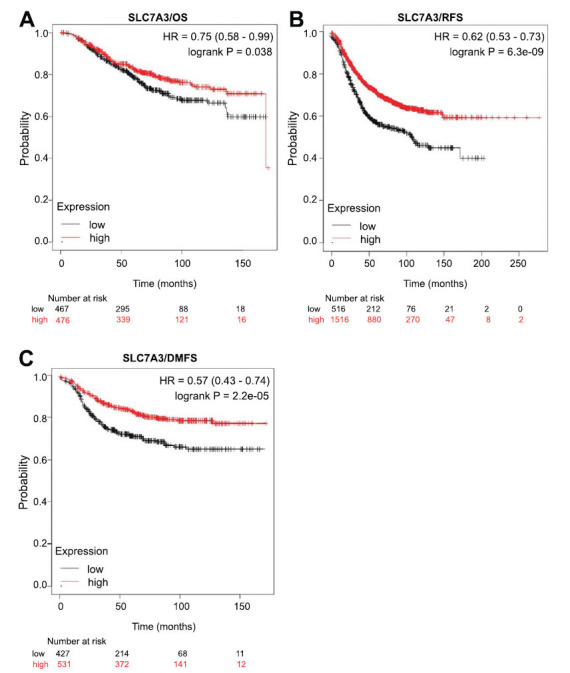
Survival curve evaluating the prognostic value of SLC7A3. (**A**) Survival curves using the Kaplan-Meier plotter database are shown for distant metastasis-free survival (DMFS), (**B**) recurrence-free survival (RFS), and (**C**) overall survival (OS).

To gain insights into the prognostic value of SLC7A3 in breast cancer, we utilized the KM-plotter online database to investigate the relationship between SLC7A3 mRNA expression and patient survival. Interestingly, low SLC7A3 expression was associated with poor relapse-free survival (RFS), poor distant metastasis-free survival (DMFS), and a trend towards poor overall survival (OS) in breast cancer patients (Fig. **5A**-**C**). Among different tumor grades, reduced SLC7A3 expression was linked to poor RFS in grade 1 breast cancer patients (Fig. **S1A**). In ER-positive breast cancer patients, higher SLC7A3 expression correlated with better RFS (Fig. **S1A**). Additionally, we observed a significant association between SLC7A3 expression and improved RFS in lymph node-positive, HER2-negative, and luminal B breast cancer patients. High SLC7A3 expression was also linked to better RFS in breast cancer patients who received chemotherapy, while decreased SLC7A3 levels corresponded to poor OS in mesenchymal stem-like breast cancer patients (Fig. **S1B**). These results suggest that SLC7A3 could act as a suppressor of tumor growth in breast cancer, and low SLC7A3 expression could serve as a marker of poor prognosis in mesenchymal stem-like breast cancer.

### 3.5 Identification of SLC7A3-interacting Genes and Proteins

**Fig. (6):**
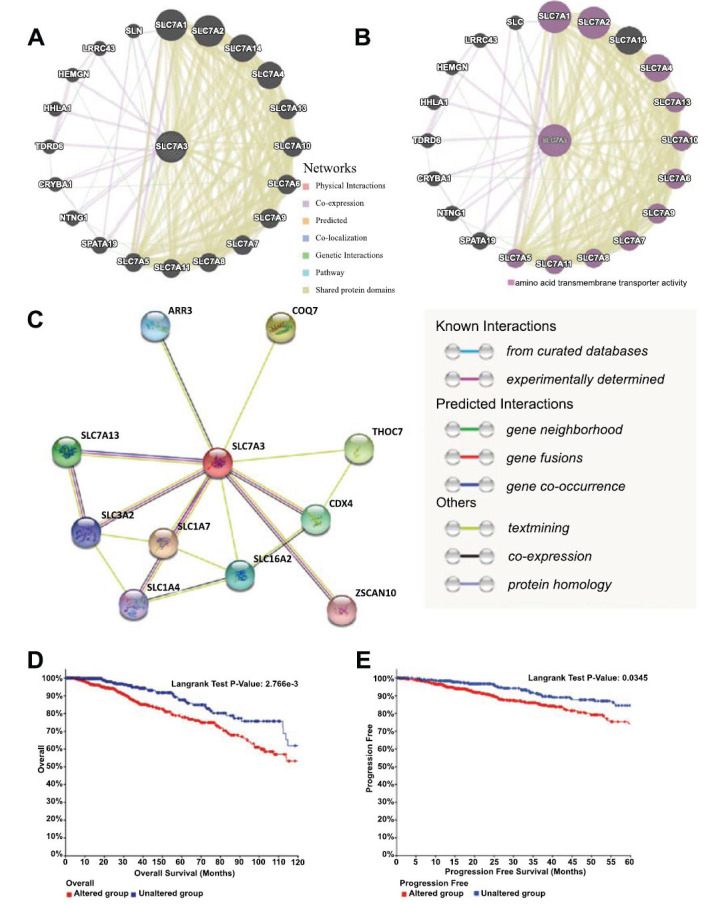
(**A** and **B**) The gene-gene interaction network for SLC7A3 was constructed using GeneMANIA. (**C**) The PPI network of SLC7A3 was generated using STRING. (**D** and **E**) Survival curves using the cBioPortal database are shown for RFS and OS.

We employed GeneMANIA to construct a gene-gene interaction network involving the SLC7A3 gene and altered neighboring genes. The analysis revealed that twenty genes with the highest altered frequencies were closely associated with SLC7A3. These genes include SLN, LRRC43, HEMGN, HHLA1, TDRD6, CRYBA1, NTNG1, SPATA19, and other members of the SLC family (Fig. **6A**). Functional analysis further demonstrated that these genes were related to L-type cationic amino acid transmembrane transporter activity (Fig. **6B**). Subsequently, a protein-protein interaction (PPI) network diagram for SLC7A3 was generated using the online STRING database, with 10 nodes representing proteins and 17 edges representing protein-protein associations (Fig. **6C**). The nodes included ARR3, COQ7, THOC7, CDX4, ZSCAN10, SLC7A13, SLC3A2, SLC1A4, SLC1A7, and SLC16A2 (Fig. **6C**).

### 3.6 Genetic Changes of SLC7A3

The frequency of SLC7A3 mutations in breast cancer was analyzed using cBioPortal on 1084 patients in a breast cancer dataset (Breast Invasive Carcinoma, TCGA, Pan-Cancer Atlas). The rate of low mRNA expression of SLC7A3 was 94.12%, 69.36%, 67.53%, and 33.83% in this dataset in breast invasive mixed mucinous carcinoma, breast invasive ductal carcinoma, breast invasive carcinoma and breast invasive lobular carcinoma, respectively (Fig. **S2**). The results of the Kaplan-Meier plotter and log-rank test showed that there was a statistically significant difference in OS and PFS between breast cancer patients with or without low SLC7A3 mRNA levels (Fig. **6D** and **E**). The above results indicated that the reduction of SLC7A3 could be a factor leading to poor prognosis of breast cancer patients. To analyze the frequency of SLC7A3 mutations in breast cancer, we used cBioPortal and examined 1084 patients in a breast cancer dataset (Breast Invasive Carcinoma, TCGA, Pan-Cancer Atlas). The low mRNA expression of SLC7A3 was 94.12%, 69.36%, 67.53%, and 33.83% in breast invasive mixed mucinous carcinoma, breast invasive ductal carcinoma, breast invasive carcinoma, and breast invasive lobular carcinoma, respectively (Fig. **S1**). The Kaplan-Meier plotter and log-rank test results indicated a statistically significant difference in overall survival (OS) and progression-free survival (PFS) between breast cancer patients with low and those without low SLC7A3 mRNA levels (Fig. **6D** and **E**). These results suggest that the reduction of SLC7A3 could be a factor contributing to poor prognosis in breast cancer patients.

### 3.7 Gene Ontology (GO) and Kyoto Encyclopedia of Genes and Genomes (KEGG) Pathway Analysis of SLC7A3 and its Co-expressed Genes in Breast Cancer

**Fig. (7):**
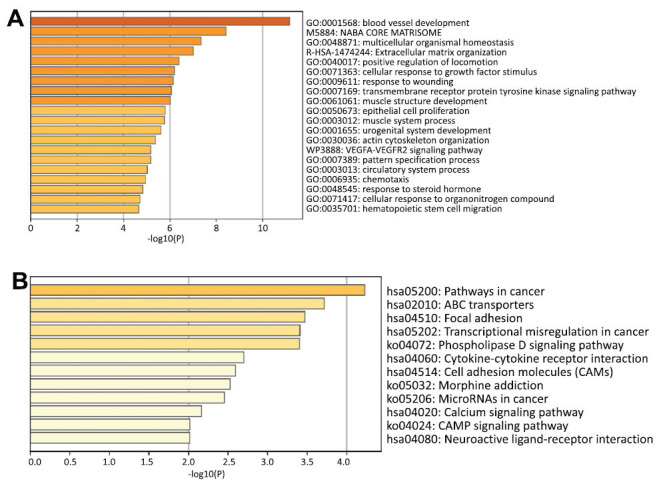
GO and KEGG enrichment analysis for SLC7A3. **(A)** Top 20 GO enrichment terms in breast cancer. **(B)** Top 12 KEGG enrichment pathways in breast cancer. SLC7A3 inhibits the proliferation and invasion of breast cancer cells *in vitro*.

We conducted data mining from TCGA dataset to identify genes that were positively or negatively co-expressed with SLC7A3. We selected a total of 185 genes that exhibited positive associations with SLC7A3 for GO enrichment analysis to explore SLC7A3-related pathways and biological functions (Fig. **S3A**-**D**). We investigated the correlations between SLC7A3 and associated genes (Fig. **7A**), SLC7A3 exhibited positive correlations with 185 genes, while no negative correlations were observed. The GO enrichment analysis revealed the top 20 significant terms for biological process, molecular function, and cellular component (Fig. **7B**), highlighting the importance of SLC7A3 in blood vessel development, integrin binding, and the collagen-containing extracellular matrix. Moreover, KEGG pathway analyses, based on 20 genes using GeneMANIA, demonstrated the significance of SLC7A3 in pathways related to cancer, ABC transporters, and focal adhesion (Fig. **7A**). These findings suggest that SLC7A3-associated genes are associated with cancer development in breast cancer.

**Fig. (8):**
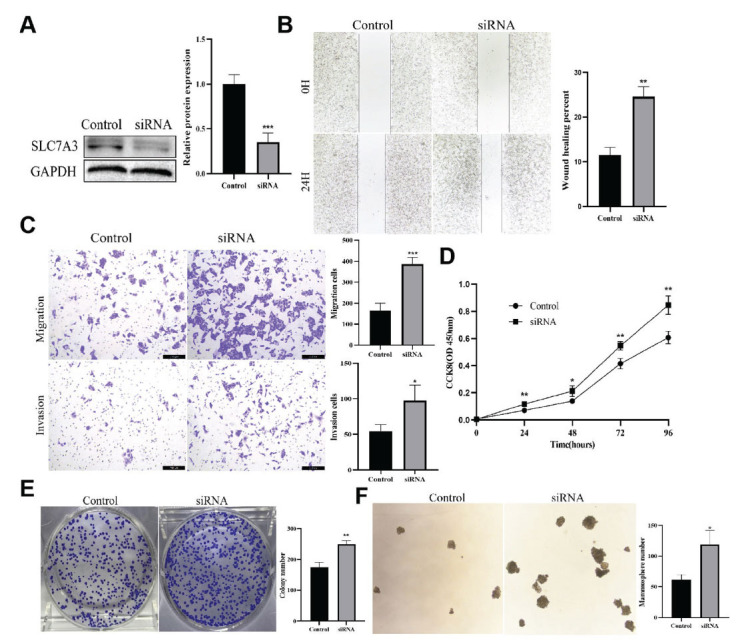
SLC7A3 inhibits the migration and invasion of breast cancer cells *in vitro*. **(A)** Expression of SLC7A3 in breast cancer cells analyzed by Western blotting after transfection. **(B)** SLC7A3 knockdown increases MCF-7 cell migration in a wound healing assay. **(C)** Migration and invasion of transfected MCF-7 cells were evaluated using transwell cell invasion and migration assay. **(D)** CCK-8 assay shows SLC7A3 knockdown promotes MCF-7 cell proliferation. **(E)** Colony formation was increased in SLC7A3 knockdown cells. **(F)** Mammosphere formation assay in control and SLC7A3-knockdown MCF-7 cells. Control: scramble small interfering RNA. SiRNA: SLC7A3 small interfering RNA. *p<0.05, **p<0.01, ***p<0.001.

SLC7A3 was knocked down in MCF-7 and MDA-MB-231 cells *in vitro*. The knockdown efficiency was shown in Fig. (**8A**), Fig. (**S4A** and **B**), and **4A**. The wound healing assay revealed that SLC7A3 knockdown promoted cell migration in both MCF-7 luminal breast cancer and MDA-MB-231 triple-negative breast cancer cells (Figs. **8B**, **S5A** and **B**). Transwell assay demonstrated increased migration and invasive ability following SLC7A3 knockdown in MCF-7 cells (Fig. **8C**), while no significant difference was observed in MDA-MB-231 cells (Fig. **S5C**). Moreover, cell proliferation assays showed a significant increase in the SLC7A3 knockdown group compared to the control group (Figs. **8D** and **S5D**). Enhanced cell colony formation was also observed after SLC7A3 knockdown in both MCF-7 and MDA-MB-231 cells (Figs. **8E** and **S5E**). Furthermore, tumor stemness assays demonstrated that SLC7A3 knockdown in breast cancer cells increased their capacity for spheroid formation and the number of spheroids, indicating enhanced mammosphere formation (Figs. **8F** and **S5F**). These results strongly suggest that SLC7A3 plays a critical role in the proliferation and invasion of breast cancer cells.

## 4 DISCUSSION

Early detection, diagnosis, and treatment are crucial in reducing breast cancer mortality. Timely diagnosis not only leads to high survival rates but also helps to minimize the economic burden associated with breast cancer [31]. Mammography and ultrasound, while valuable, are not sufficiently sensitive for early lesion detection or assessing breast cancer risk in asymptomatic individuals [32]. Utilizing blood biomarkers for breast cancer screening and early diagnosis, along with identifying breast cancer susceptibility genes in high-risk populations, can improve the early detection rate of breast cancer [33, 34].

In this study, we conducted a comprehensive bioinformatics analysis using the TIMER, Oncomine, UALCAN, and GEPIA public databases to explore the expression of SLC7A3 in breast cancer compared to normal breast tissue. Our results consistently demonstrate that SLC7A3 expression is lower in breast cancer tissues than in normal breast tissues. Moreover, SLC7A3 is expressed at a low level in both breast and pancreatic cancers compared to normal tissues. It has been reported that patients with pancreatic cancer carry rare germline deleterious variants of breast cancer susceptibility genes such as BRCA1, BRCA2, PALB2, and ATM [35]. However, the examined pancreatic cancer susceptibility variants have not been individually associated with breast cancer risk. Our comprehensive examination of SLC family constituents reveals a notable association between SLCs and breast cancer over the past decade. Among these, a Chinese patent elucidates a detection kit for detecting postoperative breast cancer recurrence and metastasis involving SLC50A1, thus introducing SLC50A1 as a biomarker for identifying postoperative recurrence or metastasis in breast cancer cases [15]; a New Zealand patent divulges various early diagnostic markers for breast cancer, including SLC1A5 and SLC7A5 [16]. In our comprehensive literature search through PubMed, we found eight amino acid transporters from SLC7A1 to SLC7A14 have not been studied in breast cancer. Consequently, we investigated the expression of these transporters and their correlation with prognosis in breast cancer patients using the UALCAN online database. Notably, only SLC7A3 and SLC7A4 showed significant associations with breast cancer patient prognosis. In terms of breast cancer classification, all subtypes of breast cancer exhibit lower SLC7A3 expression compared to normal breast tissue, especially TNBC and HER2-positive breast cancer, which are aggressive breast cancers.

Recent studies have suggested that white women have lower breast cancer incidence and risk of death compared to women of other racial backgrounds [36]. Our findings align with this observation, as Caucasian breast cancer patients displayed higher levels of SLC7A3 than their African-American and Asian counterparts. This difference in SLC7A3 expression may partially explain the high incidence of breast cancer rates in Asian countries compared to Western countries in recent decades. SLC7A3 functions as a sodium-independent cationic amino acid transporter, and its expression is higher in normal breast tissue than in breast cancer tissue. Kaplan-Meier analyses in various databases consistently demonstrate that higher SLC7A3 expression at diagnosis is associated with better prognosis of breast cancer patients. This observation contrasts with a previous report showing that papillary thyroid carcinoma patients with high SLC7A3 expression have poor survival rates and another study indicating that SLC7A3 promotes tumor growth by increasing intracellular arginine to maintain mTORC1 activation [37]. Our functional experiments, however, indicate that SLC7A3 functions as a cancer suppressor gene, inhibiting the progression of breast cancer. One possibility is that SLC7A3 levels may be related to the TP53 tumor suppressor pathway, especially in the context of glutamine deprivation [38]. However, our data show that SLC7A3 tumor suppressor function appears to be independent of TP53 status.

The prognostic significance of SLC7A3 in breast cancer patients was thoroughly investigated. Forest plot analysis indicated that SLC7A3 expression is correlated with relapse-free survival in grade 1 but not in grades 2 and 3 breast cancer. The box plot showed no significant difference in SLC7A3 expression between grade 2 and 3 patients. The cBioPortal database analysis revealed that approximately 80% of breast invasive ductal carcinoma patients have low SLC7A3 mRNA expression levels, suggesting that reduced SLC7A3 gene transcription may contribute to poor prognosis in this subset of patients. Kaplan-Meier analysis further supports a closer association between SLC7A3 expression and survival, indicating that higher SLC7A3 expression is predictive of a better prognosis for breast cancer patients. Mesenchymal stem-like cells have been implicated in tumor development, increasing the motility, invasiveness, and metastatic potential of breast cancer cells [39, 40]. Our forest plot indicates that SLC7A3 expression is positively correlated with overall survival in mesenchymal stem-like breast cancer, reinforcing the notion that SLC7A3 acts as an independent tumor suppressor in breast cancer. Gene enrichment analysis demonstrates that SLC7A3-associated genes mainly belong to the SLC7 family, which includes cationic amino acid/glycoprotein transporters essential for amino acid nutrition and tumor cell survival [38]. We found that these genes are enriched in tumor-related pathways, suggesting their possible correlation with tumor progression. Nitric oxide synthase (NOS) has been found in various tumors, and NO can regulate tumor occurrence and development [38]. Our research suggests that in late-stage breast cancer, SLC7A3 may play a pro-tumorigenic role. NO can convert anti-tumor M1 macrophages into pro-tumor M2 macrophages [41]. This may partially explain the poor prognosis associated with high expression of SLC7A3 in late-stage tumors observed in other studies [37].

Targeting SLC7A3 to inhibit arginine uptake is suggested as a potential therapeutic strategy, especially in the context of osteosarcoma, as SLC7A3 facilitates arginine uptake, which in turn could promote tumor growth and metastasis [38, 42]. SLC7A3 is induced early by glutamine deprivation to promote cell growth through the maintenance of mTORC1 caused by arginine influx. The early transcriptional effect by p53 in response to glutamine deprivation is seen as a pro-survival response, with SLC7A3 playing a crucial role in this adaptive mechanism [38]. These findings collectively hint at the potential of SLC7A3 as a target in cancer treatment strategies, especially through its involvement in amino acid transport and interaction with signaling pathways like mTORC1. The modulation of SLC7A3 activity could impact tumor growth, metastasis, and cellular adaptive responses to metabolic stress, thus opening avenues for therapeutic interventions.

Our analysis indicates an association between high SLC7A3 expression in breast cancer patients and an increased likelihood of lymph node metastasis. This trend may stem from the relatively fewer N3 patients in our study compared to N0-2 cases. Additionally, it could potentially be attributed to shifts in the tumor microenvironment, facilitating enhanced lymphatic entry of tumor cells. This scenario underscores the intricate dual role that SLC7A3 plays in breast cancer development, likely shaped by its interactions with diverse signaling pathways and molecules, thereby engendering varied biological outcomes. This study bears certain sample biases, particularly the disproportionately low number of N3 patients relative to N0-2 cases, which might skew the analysis regarding the association between SLC7A3 expression and lymph node metastasis. Such biases could potentially compromise the accuracy and generalizability of the findings. In conclusion, further research is warranted to elucidate the mechanistic underpinnings of SLC7A3 in breast cancer.

## 5 CURRENT & FUTURE DEVELOPMENTS

This study elucidates the impact of SLC7A3 on the biological attributes of breast cancer cells and the survival prognosis of patients. Our data underscores SLC7A3's function as a tumor suppressor gene in breast cancer. Subsequent cell experiments are imperative to authenticate the involvement of SLC7A3 in the tumorigenesis and progression of breast cancer, while animal experiments are warranted to establish SLC7A3's viability as a therapeutic target for breast cancer.

## CONCLUSION

This study unveils a tumor suppressor role of SLC7A3 in breast cancer. Our findings contribute to a better understanding of the role of SLC7A3 and its potential applications in breast cancer diagnosis and prognosis.

## Figures and Tables

**Fig. (1):**
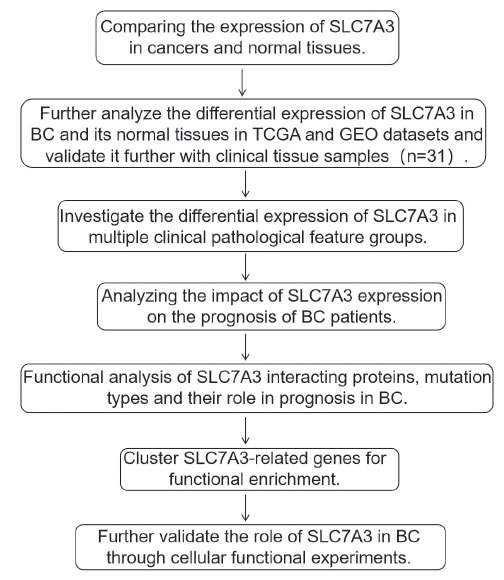
Research design.

**Table 1:** SLC7A3 expression in breast cancer.

**Cancer Subtype**	***P*-value**	**Fold Change**	**Rank (%)**	**Sample**
Invasive ductal breast carcinoma stroma	5.06E-07	-9.794	10	22
Mucinous breast carcinoma	1.02E-07	-2.589	10	65
Invasive breast carcinoma	2.06E-11	-2.147	10	137
Ductal breast carcinoma	5.08E-05	-5.654	10	47

## Data Availability

The data that support the findings of this study are openly available in these online databases listed in Material and Methods. These data were derived from TCGA, GEO and GTEx public databases.

## LIST OF ABBREVIATIONS

DFS: Disease-Free Survival
DMEM: Dulbecco's Modified Eagle's Medium
EGF: Epidermal Growth Factor
ER: Estrogen Receptor
FBS: Fetal Bovine Serum
GEPIA: Gene Expression in Profiling Interactive Analysis
HER2: Human Epidermal Growth Factor Receptor 2
IHC: Immunohistochemistry
NO: Nitric Oxide
OS: Overall Survival
SLC7A3: Solute Carrier Family 7 Member 3
TCGA: The Cancer Genome Atlas

## STANDARDS OF REPORTING

STROBE guidelines were followed.
