# Supplementary material for: Increased SLC7A3 Expression Inhibits Tumor Cell Proliferation and Predicts a Favorable Prognosis in Breast Cancer
Source: Recent Pat Anticancer Drug Discov. 2024 Jan 8;20(1):55–70. doi: 10.2174/0115748928279007231130070056 (PMC11826905; doi:10.2174/0115748928279007231130070056)
Supplement: Supplementary file 1 [file PRA-20-1-55_SD1.pdf]

SUPPLEMENTARY MATERIAL

Increased SLC7A3 Expression Inhibits Tumor Cell Proliferation and Predicts a Favorable Prognosis in Breast Cancer

Lifang He[1,2,#], Yue Xu[1,#], Jiediao Lin[1], Stanley Li Lin[1,3*,#] and Yukun Cui[1,*,#]

[1]Guangdong Provincial Key Laboratory for Breast Cancer Diagnosis and Treatment, Cancer Hospital of Shantou University Medical College, Shantou, Guangdong Province, 515000, China; [2]Breast Center, Cancer Hospital of Shantou University Medical College, Shantou, Guangdong Province, 515000, China; [3]Department of Cell Biology and Genetics, Shantou University Medical College, Shantou, Guangdong Province, 515000, China

STROBE Statement—checklist of items that should be included in reports of observational studies

	Item No.	Recommendation	Page No.	Relevant text from manuscript
**Title and abstract**	1	(*a*) Indicate the study's design with a commonly used term in the title or the abstract	1-2	***Objective:*** We aimed to identify the molecule that governs arginine metabolism in breast cancer and to elucidate its significance. ***Methods:*** We analyzed the correlation between the expression of solute carrier family 7 member 3 (SLC7A3), the major arginine transporter, and breast cancer survival in various databases, including GEPIA, UALCAN, Metascape, String, Oncomine, KM-plotter, CBioPortal and Prognosis. Additionally, we validated our findings through bioinformatic analyses and experimental investigations, including colony formation, wound healing, transwell, and mammosphere formation assays.
		(*b*) Provide in the abstract an informative and balanced summary of what was done and what was found	1-2	***Results:*** Our analysis revealed a significant reduction in SLC7A3 expression in all breast cancer subtypes compared to adjacent breast tissues. Kaplan-Meier survival analyses

				demonstrated that high SLC7A3 expression was positively associated with decreased nodal metastasis (HR=0.70, 95% CI [0.55, 0.89]), ER positivity (HR=0.79, 95% CI [0.65, 0.95]), and HER2 negativity (HR=0.69, 95% CI [0.58, 0.82]), and increased recurrence-free survival. Moreover, low SLC7A3 expression predicted poor prognosis in breast cancer patients for overall survival. Additionally, knockdown of SLC7A3 in MCF-7 and MDA-MB-231 cells resulted in increased cell proliferation and invasion in vitro. ***Conclusion:*** Our findings indicate a downregulation of SLC7A3 expression in breast cancer tissues compared to adjacent breast tissues. High SLC7A3 expression could serve as a prognostic indicator for favorable outcomes in breast cancer patients due to its inhibitory effects on breast cancer cell proliferation and invasion.
Introduction				
Background/rationale	2	Explain the scientific background and rationale for the investigation being reported	3-4	The precise mechanisms underlying the association between SLC7A3 expression and survival outcomes in breast cancer patients are yet to be thoroughly explored. There's a call for further studies to verify the diagnostic and prognostic value of SLC7A3 in breast cancer, indicating a need for more comprehensive research to substantiate initial findings[15].In this study, we explored the diagnostic and prognostic value of SLC7A3 in breast cancer.

Objectives	3	State specific objectives, including any prespecified hypotheses	3-4	In this study, we explored the diagnostic and prognostic value of SLC7A3 in breast cancer.
Methods				
Study design	4	Present key elements of study design early in the paper	5	Fig.1
Setting	5	Describe the setting, locations, and relevant dates, including periods of recruitment, exposure, follow-up, and data collection	5-11	This research received approval from the Research Ethics Committee of the Cancer Hospital affiliated with Shantou University Medical College. Prior to the study, written informed consents were obtained from all participants. For immunohistochemical staining, a total of thirty-one cases of formalin-fixed, paraffin-embedded breast cancer tissues, and adjacent breast tissues were used.
Participants	6	***Cohort study***—Give the eligibility criteria, and the sources and methods of selection of participants. Describe methods of follow-up<br>***Case-control study***—Give the eligibility criteria, and the sources and methods of case ascertainment and control selection. Give the rationale for the choice of cases and controls<br>***Cross-sectional study***—Give the eligibility criteria, and the sources and methods of selection of participants	N/A	
		Cohort study—For matched studies, give matching criteria and number of exposed and unexposed<br>***Case-control study***—For matched studies, give matching criteria and the number of controls per case	N/A	
Variables	7	Clearly define all outcomes, exposures, predictors, potential confounders, and effect modifiers. Give diagnostic criteria, if applicable	8	The immunohistochemical staining results were analyzed and scored by two pathologists who were unaware of the sources of the clinical samples. Staining intensity was evaluated using a semi-quantitative integration method, with intensity scores assigned as follows: 0 for negative, 1 for weak, 2 for moderate, and 3 for strong. The frequency of positive cells was categorized as follows: 0 for less than 5%, 1 for 5% to 25%, 2 for 26% to

				50%, 3 for 51% to 75%, and 4 for greater than 75%. A combined "histoscore" was calculated as the result of multiplying the average staining intensity (0–3) by the average percentage of positive cells (0–4), with a maximum possible score of 12. The degree of SLC7A3 staining was determined by summing the staining intensity and the positive cell percentage scores, establishing levels of low expression (0–6) and high expression.
Data sources/ measurement	8*	For each variable of interest, give sources of data and details of methods of assessment (measurement). Describe comparability of assessment methods if there is more than one group	8	The immunohistochemical staining results were analyzed and scored by two pathologists who were unaware of the sources of the clinical samples. Staining intensity was evaluated using a semi-quantitative integration method, with intensity scores assigned as follows: 0 for negative, 1 for weak, 2 for moderate, and 3 for strong. The frequency of positive cells was categorized as follows: 0 for less than 5%, 1 for 5% to 25%, 2 for 26% to 50%, 3 for 51% to 75%, and 4 for greater than 75%. A combined "histoscore" was calculated as the result of multiplying the average staining intensity (0–3) by the average percentage of positive cells (0–4), with a maximum possible score of 12. The degree of SLC7A3 staining was determined by summing the staining intensity and the positive cell percentage scores, establishing levels of low expression (0–6) and high expression.

Bias	9	Describe any efforts to address potential sources of bias	11-12	A correlation heat map depicting the relationship between SLC7A3 and its associated genes was generated using data from the UALCAN database, with Spearman correlation analysis. A p-value of less than 0.05 was considered statistically significant. All experiments were conducted three times, with samples measured in duplicate or triplicate.
Study size	10	Explain how the study size was arrived at	7-8	This research received approval from the Research Ethics Committee of the Cancer Hospital affiliated with Shantou University Medical College. Prior to the study, written informed consents were obtained from all participants. For immunohistochemical staining, a total of thirty-one cases of formalin-fixed, paraffin-embedded breast cancer tissues, and adjacent breast tissues were used.(Approval Number: 201811).

*Give information separately for cases and controls in case-control studies and, if applicable, for exposed and unexposed groups in cohort and cross-sectional studies.

A

Subgroup	Number	Hazard Ratio(95%CI)-RFS	p-value
**ER status**			
Positive	5526	0.79(0.65,0.95)	0.012
negative	2009	1.02(0.79,1.31)	0.88
**PR status**			
Positive	1559	0.73(0.5,1.07)	0.1
negative	1989	0.79(0.56,1.1)	0.16
**HER 2 status**			
Positive	1273	0.78(0.57,1.05)	0.1
negative	6262	0.69(0.58,0.82)	2.50E-05
**Subtype-PAM50**			
Normal	309	0.78(0.29,2.1)	0.62
Basal	1671	0.9(0.66,1.22)	0.49
Luminal A	2470	0.81(0.57,1.15)	0.24
Luminal B	2005	0.72(0.56,0.94)	0.014
HER2+	1080	0.86(0.62,1.2)	0.38
**Lymph node status**			
Positive	2153	0.7(0.55,0.89)	0.004
negative	2829	0.77(0.53,1.13)	0.18
**Grade**			
1	576	0.24(0.07,0.85)	0.016
2	1795	1.07(0.65,1.76)	0.78
3	2058	0.9(0.67,1.21)	0.49
**TP53 status**			
Mutated	272	0.72(0.4,1.3)	0.27
Wild type	388	1.43(0.61,3.35)	0.4
**TNBC subtype**			
Basal like1	418	0.88(0.5,1.56)	0.66
Basal like2	165	0.7(0.34,1.47)	0.35
Immunomodulatory	462	1.96(0.86,4.43)	0.1
Mesenchymal	382	0.77(0.45,1.3)	0.32
Mesenchymal stem-like	201	0.52(0.2,1.39)	0.19
Luminal androgen receptor	413	0.9(0.54,1.51)	0.69
**Systemically untreated patients**			
Yes	1030	1.08(0.39,2.97)	0.89
no	3733	0.56(0.45,0.7)	1.20E-07
**Endocrine**			
Yes	1813	0.81(0.51,1.29)	0.38
No	2038	1.02(0.66,1.57)	0.94
tamoxifen	1063	0.74(0.3,1.84)	0.51
**Chemotherapy**			
Yes	2342	0.31(0.14,0.66)	0.0013
no	2105	0.9(0.47,1.72)	0.76

B

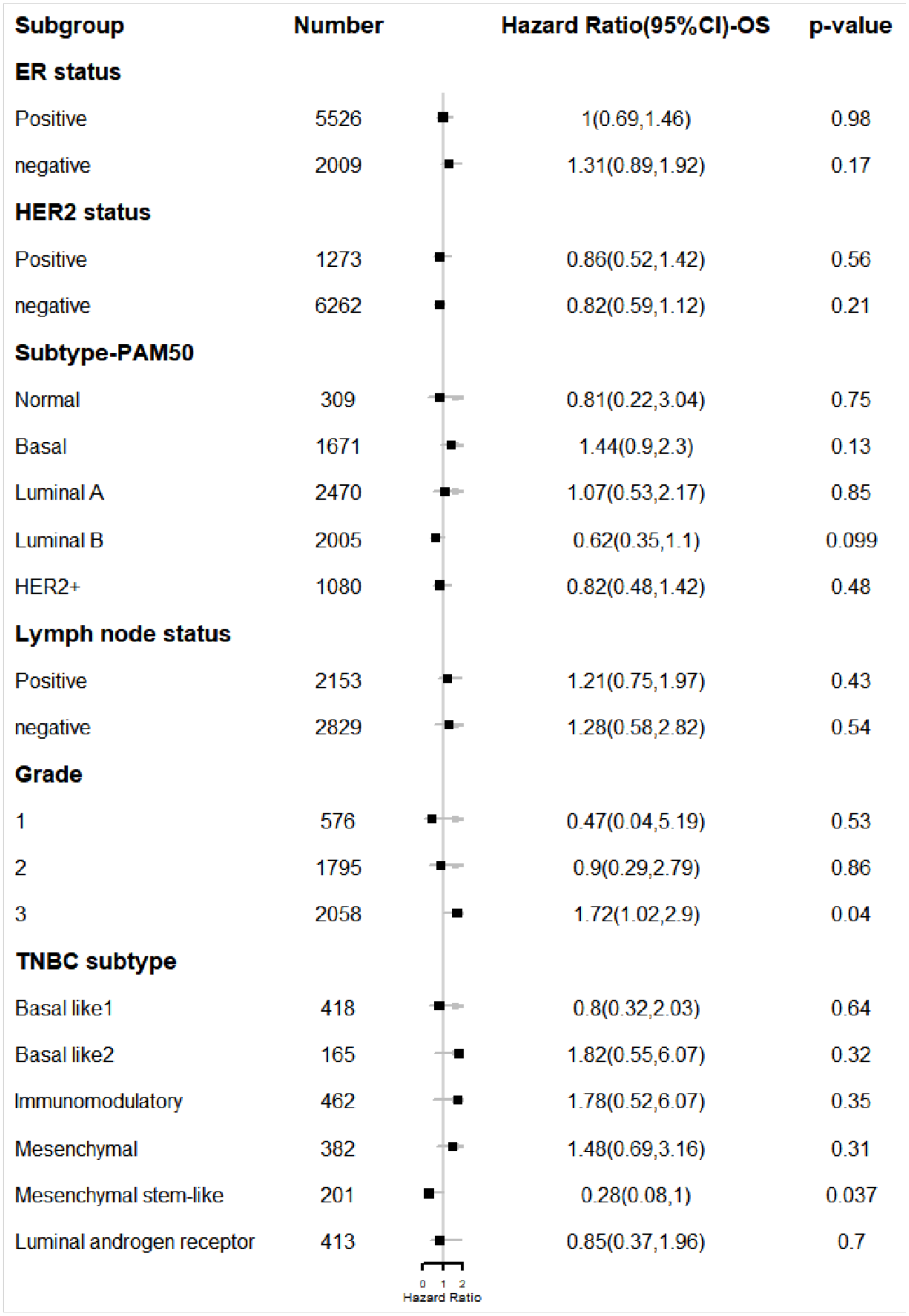

Supplementary Figure 1 Forest plot shows the correlation between SLC7A3 expression and clinicopathological parameters in breast cancer patients. (A) The correlation between SLC7A3 expression and clinicopathological parameters in breast cancer patients based on RFS and (B) OS.

A

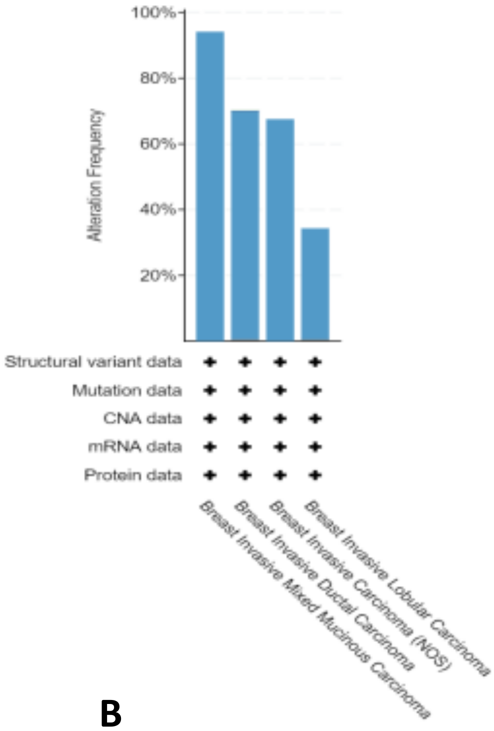

B

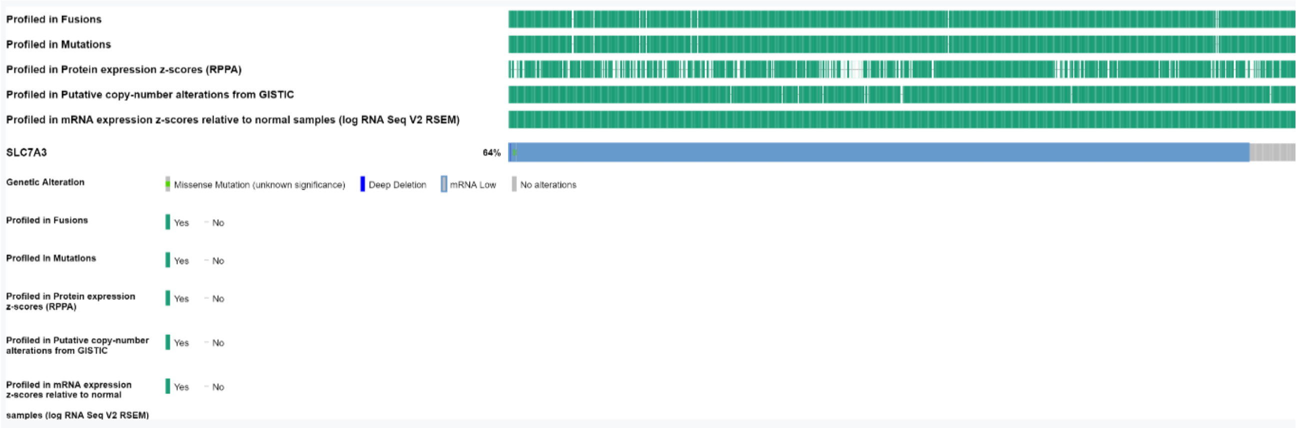

Supplementary Figure 2 (A) SLC7A3 mRNA mutation frequency in different breast cancer subtypes are shown. (B) SLC7A3 mutation in details are shown.

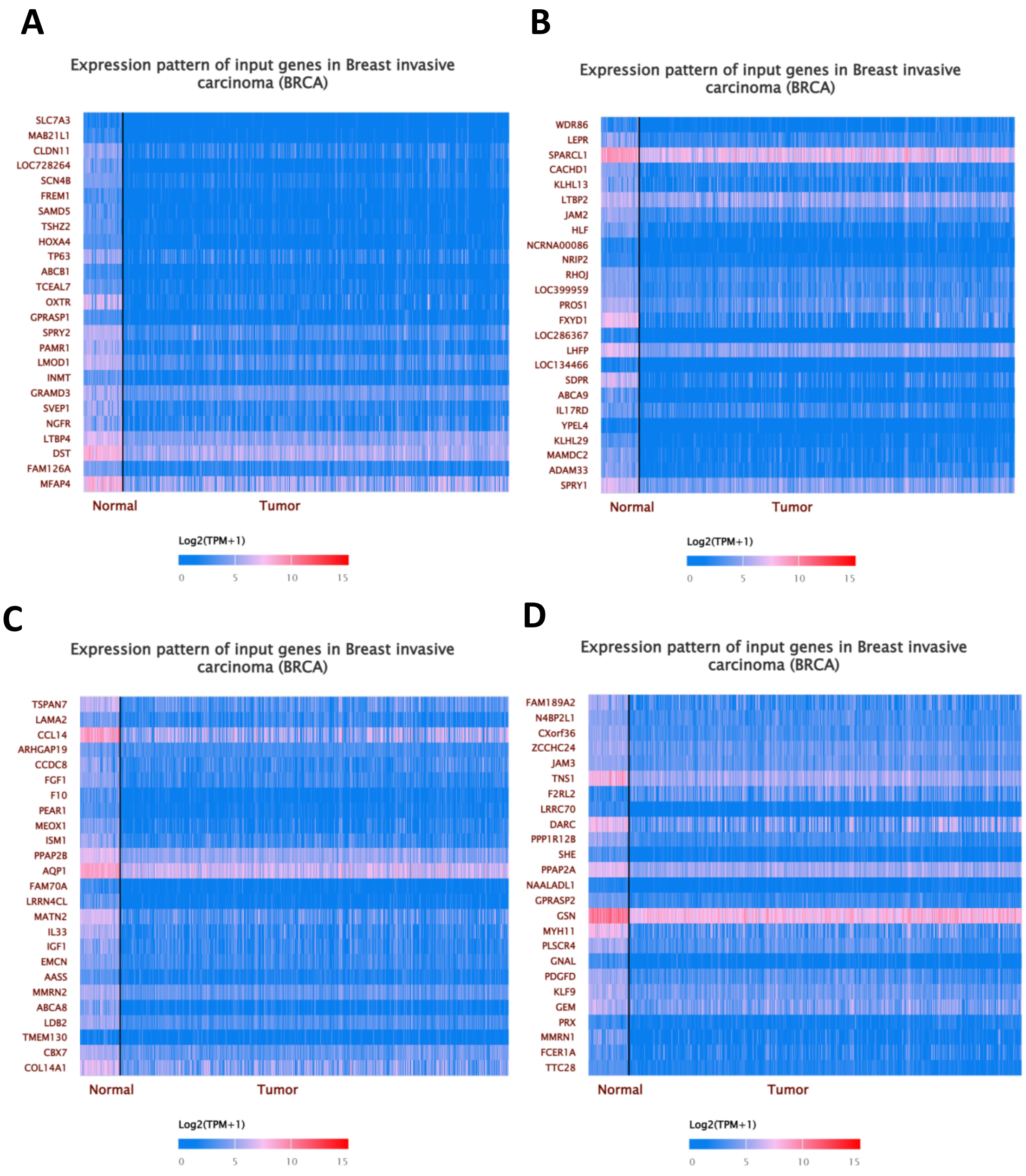

Supplementary Figure 3 (A-D) Heat maps showing the top 100 differencially expressed genes associated with SLC7A3 in breast cancer and normal tissues based on TCGA database.

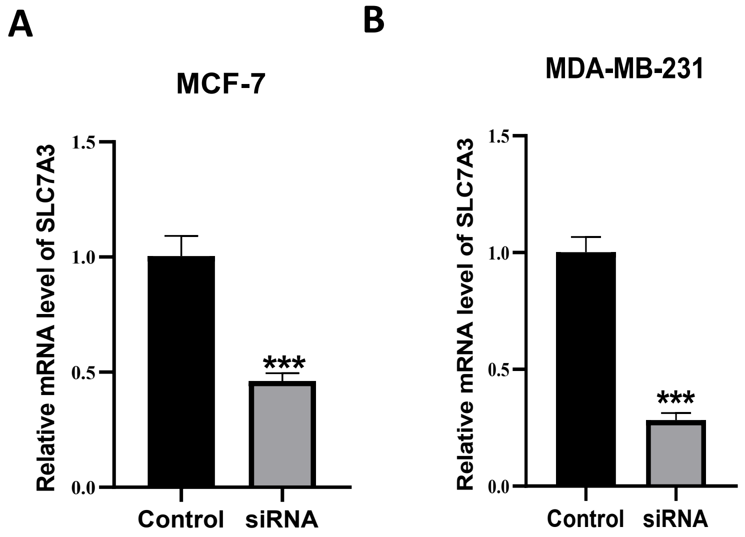

Supplementary Figure 4 (A) mRNA expression level SLC7A3 in MCF-7 and (B) MDA-MB-231 cells after SLC7A3 knockdown. ***P<0.001

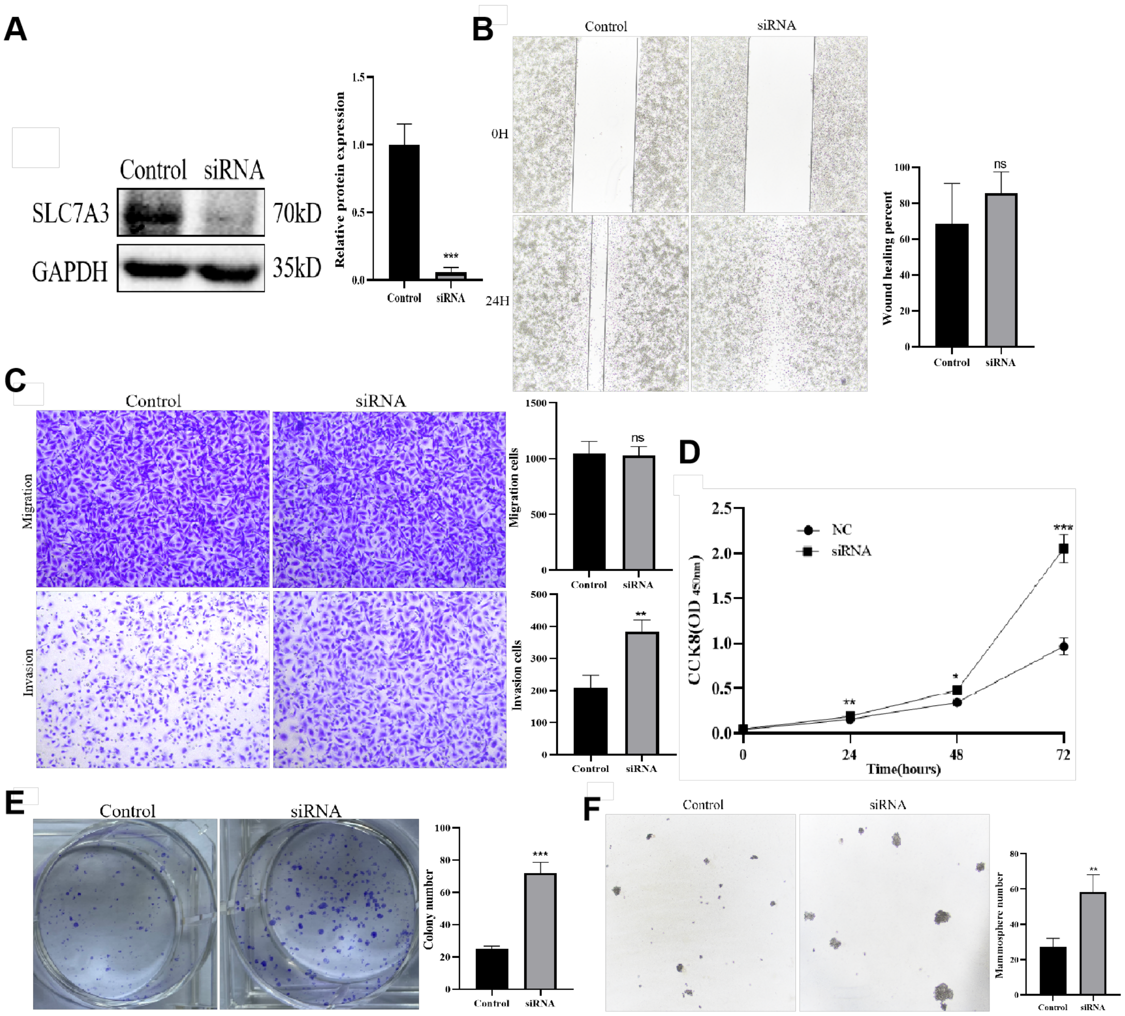

Supplementary Figure 5 | SLC7A3 inhibited the proliferation and invasion of breast cancer cells in vitro. (A) Expression of SLC7A3 in MDA-MB-231 cells was analyzed by western blot after transfection. (B) SLC7A3 knockdown increases MDA-MB-231 cells migration in a wound healing assay. (C) Migration and invasion of transfected MDA-MB-231 cells was evaluated using transwell cell invasion and migration assays. (D) CCK-8 assay shows SLC7A3 knockdown enhances MDA-MB-231 cell proliferation. (E) SLC7A3 knockdown increases colony formation. (F) Mammosphere formation assays in control and SLC7A3-knockdown MDA-MB-231 cells. Control: scramble small interfering RNA. SiRNA: SLC7A3 small interfering RNA. *p<0.05, **p<0.01, ***p<0.001.

Supplementary Table 1 Primer sequence for RT-PCR.

primers ID	Primer squence (5'-3')
SLC7A3-Forward	ATTTGCTTTCTCCGAGGGCA
SLC7A3-Reverse	ATACCCAGCTCCAACACACG
ACTIN-Forward	CATGTACGTTGCTATCCAGGC
ACTIN-Reverse	CTCCTTAATGTCACGCACGAT

Supplementary Table 2 siRNA sequence.

**Negative Control**	
sense(5'-3')	UUCUCCGAACGUGUCACGUTT
antisense(5'-3')	ACGUGACACGUUCGGAGAATT
**SLC7A3-Homo**	
sense(5'-3')	GCGAGGUGGCCAAAGAUAATT
antisense(5'-3')	UUAUCUUUGGCCACCUCGCTT

DISCLAIMER: The above article has been published, as is, ahead-of-print, to provide early visibility but is not the final version. Major publication processes like copyediting, proofing, typesetting and further review are still to be done and may lead to changes in the final published version, if it is eventually published. All legal disclaimers that apply to the final published article also apply to this ahead-of-print version.
